# Does the increase in health insurance benefits have different effects on the health of middle-aged and older people individuals in rural areas? Analysis based on quantile difference-in-differences method

**DOI:** 10.3389/fpubh.2024.1322790

**Published:** 2024-04-15

**Authors:** Cheng Qin, Xueyi Wang

**Affiliations:** ^1^School of Economics/China-ASEAN Institute of Financial Cooperation, Guangxi University, Nanning, China; ^2^School of Business, Guangxi University, Nanning, China

**Keywords:** health insurance integration, rural, middle-aged and older people, frailty, quantile difference-in-differences

## Abstract

In the context of healthy aging, enhancing health performance is an intrinsic requirement for the development and reform of the health insurance system. This paper mainly discusses the health effects of increasing medical insurance benefits on people with different levels of health. So this paper utilizes multiple rounds of data from the China Health and Retirement Longitudinal Study (CHARLS) and employs the quantile difference-in-differences method to systematically investigate the impact effects of the integration of urban and rural residents' health insurance on the frailty levels of rural middle-aged and older people individuals. The research findings are as follows: Firstly, the integration of urban and rural resident health insurance has mitigated the frailty level of rural older people individuals, with a more pronounced impact on those with poorer health statuses. Secondly, in terms of heterogeneity analysis, the health performance effects of the urban-rural health insurance integration policy are more significant among the older people population and in the western regions. Thirdly, the integration of urban and rural resident health insurance primarily improves health by reducing the burden of medical expenses, with a greater impact on the older people population with poorer health statuses. Based on the research findings, we recommend addressing the disparities in healthcare benefits across various insurance systems, alleviating the financial burden of healthcare for impoverished individuals, and consistently improving the coordination of healthcare insurance policies for both urban and rural residents.

## 1 Introduction

The healthcare insurance system is a significant institutional arrangement aimed at safeguarding the health of residents, enhancing their wellbeing, and maintaining social harmony and stability. Especially in rural areas of developing countries, the level of healthcare for the population is relatively inadequate. In 2003, China officially launched the New Rural Cooperative health insurance System (referred to as “New Rural Cooperative health insurance”), gradually establishing a basic health insurance system for rural residents. In 2007, the Urban Resident health insurance System, primarily targeting urban informal workers, was also established, further expanding the scope of China's health insurance system. Since 2011, more than 95% of the population in China has been covered by basic health insurance. The development direction of the basic health insurance system has shifted from achieving universal coverage to ensuring fairness. However, long-standing urban-rural development disparities persist, and the contradictions within the urban-rural divide have become more pronounced during the rapid urbanization process. Differences in the design of urban and rural social security systems have greatly affected rural residents' access to medical services and healthcare. This misalignment goes against the socialist goal of promoting equal access to public services and is particularly evident in the significant disparities that still exist between the New Rural Cooperative health insurance and Urban Resident health insurance systems in terms of medical benefits. These two systems are relatively similar in terms of funding mechanisms and institutional design, providing a strong foundation for further integration.

In 2016, the State Council of China issued the “Opinions on Integrating the Basic health insurance Systems for Urban and Rural Residents,” which called for the accelerated integration of the Urban Resident Basic health insurance and the New Rural Cooperative Medical Care schemes, aiming to establish a unified basic health insurance system for both urban and rural residents. By unifying coverage, financing policies, benefit packages, the health insurance catalog, designated healthcare providers, and fund management, the goal is to achieve integrated management and services for both urban and rural resident health insurance. This will significantly improve the health insurance benefits for rural residents and is expected to enhance their overall health status. Rural older people individuals represent one of the economically most vulnerable groups, concurrently lacking older people care, medical, and caregiving services, which makes them highly susceptible to issues such as “impoverishment due to illness” and “returning to poverty due to illness.” The health status of this demographic has become crucial for rural labor force development, rural industrial revitalization, and social stability.

The Health Capital Demand model, originally formulated by Grossman, posits that individuals' demand for medical services is derived from their demand for health (1). Health insurance alters the price of medical services when individuals require them. A reduction in the price of medical services stimulates their utilization, and it is undeniable that an increase in the utilization of medical services has a positive impact on health promotion. However, early studies on the impact of health insurance on health in China yielded inconsistent results. For instance, in their analysis of urban residents in the year 2000, Zhao and Hou found that the presence or absence of health insurance did not have a significant effect on health (2). Huang and Li conducted studies in 2002 and 2005, which indicated that health insurance promoted the health of older people individuals (3). The differing results could very likely be attributed to the early stage of the health insurance system's establishment at that time, where the health effects might not have been evident yet, along with differences among the study populations. Furthermore, there is not a complete consensus within the academic community regarding the health improvement effects of the New Rural Cooperative Medical Scheme (NRCMS). Lei and Lin found that the New Rural Cooperative Medical Scheme (NRCMS) had no significant impact on the health of farmers (4). However, the results of Wu and Shen's study indicated that the NRCMS system had a positive influence on the health improvement of farmers (5). Since 2016, China has undergone a large-scale integration of urban and rural residents' health insurance, merging the New Rural Cooperative Medical Scheme (NRCMS) and the urban resident health insurance system. This integration has effectively increased the health insurance benefits for rural residents. Research on the impact of health insurance on health has evolved from examining whether residents have health insurance to assessing the influence of improvements in health insurance benefits (6). Some scholars have already examined the impact of the integration of urban and rural resident health insurance on health. Studies by Zheng et al. and Tan and Cao both indicate that the integration of urban and rural resident health insurance can enhance the utilization of medical services among the insured population, thereby promoting health (7, 8). Hong et al. went further to demonstrate that the integration of urban and rural resident health insurance not only promotes health but also reduces health losses among rural middle-aged and older people individuals (9).

However, most previous studies have typically chosen one or a few indicators among self-rated health, mental health, or cognitive impairment. While these indicators can provide relatively detailed insights into the impact of various factors, both subjective and objective, on different health states, they may not comprehensively reflect the overall health status of middle-aged and older people individuals. Secondly, previous research on the impact of health insurance, including New Rural Cooperative Medical Schemes (NRCMS) and urban-rural resident health insurance (URRMI), on health has often assumed that the effects of health insurance on middle-aged and older people individuals with different health statuses are homogeneous. However, with the increase in reimbursement rates, the impact of health insurance on the health of critically ill patients may be more significant. This is because the current health insurance system in China primarily focuses on catastrophic illnesses, particularly those requiring hospitalization, and provides additional medical assistance policies for patients with extremely severe illnesses. Moreover, the higher reimbursement rates mean that critically ill patients will receive larger compensation amounts from health insurance policies. Consequently, the marginal effect of health insurance on their health improvement will be greater. Previous research has often overlooked the heterogeneity in the impact of urban-rural resident health insurance integration on health improvement among individuals with different health statuses. Importantly, as individuals enter middle and old age, their health tends to decline significantly, and there are greater variations in the health status of different middle-aged and older people individuals. Examining the effects of urban-rural resident health insurance integration on individuals with varying health statuses can facilitate the development of more precise healthcare policies tailored to patients with severe illnesses.

## 2 Theoretical analysis

Grossman introduced the concept of health capital and developed a health demand model (1). In this model, it is assumed that consumers' demand for medical services is driven by their demand for health (1). Assuming a representative consumer's utility function at different stages of life is:


(1) $$\begin{array}{l} U=U\left(\phi_{t}H_{t},Z_{t}\right),t=0,1,\ldots ,n \end{array}$$

*H*_*t*_ represents the stock of health capital, while *Z*_*t*_ represents the quantity of goods other than health. Consumers face two types of constraints when making investment decisions: one is the income constraint, as in traditional consumer theory, and the other is the time constraint. The budget constraint that consumers face is given by:


(2) $$\begin{array}{l} \overset{n}{\underset{t=0}{\sum }}\frac{P_{t}M_{t}+Q_{t}X_{t}}{{\left(1+r\right)}^{t}}=\overset{n}{\underset{t=0}{\sum }}\frac{W_{t}TW_{t}}{{\left(1+r\right)}^{t}}+A_{0} \end{array}$$

*P*_*t*_ and *Q*_*t*_ represent the prices of medical services (Mt) and other commodities (Xt), respectively. *W*_*t*_ denotes the wage rate, *TW*_*t*_ signifies working hours, and *A*_0_ represents the initial wealth. In addition to the budget constraint, consumers also face a time constraint:


(3) $$\begin{array}{l} TW_{t}+T_{ht}+T_{t}+TL_{t}=\Omega \end{array}$$

Where *TW*_*t*_ represents working time, *TL*_*t*_ signifies time lost due to poor health, *T*_*t*_ stands for the time allocated to producing goods, and *T*_*ht*_ represents time invested in health improvement. The equilibrium conditions for the above model are derived as follows:


(4) $$\begin{array}{l} \gamma_{t}+\alpha_{t}=r+\delta_{t} \end{array}$$

**Figure 1:**
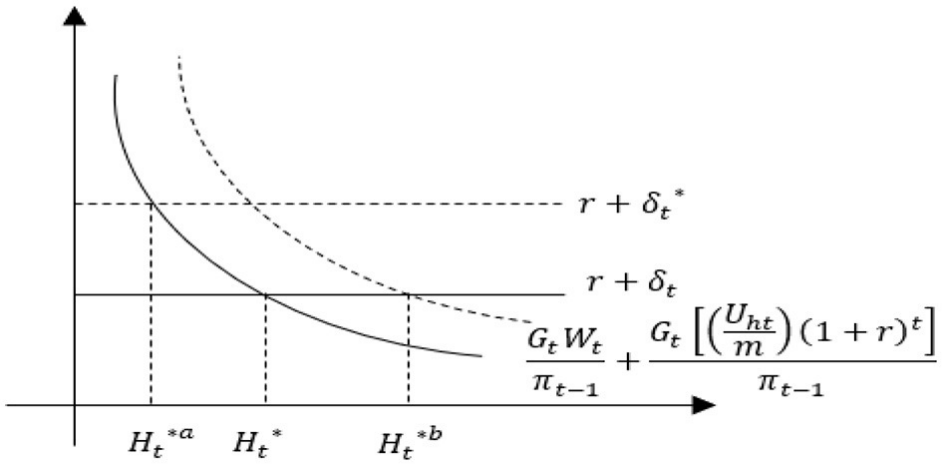
Comparative statics analysis of health demand.

γ_*t*_ represents the market rate of return on health as an investment, α_*t*_ represents the marginal utility value of health when it is consumed as a good. This is often referred to as the consumer's psychic return rate. r represents the interest rate, δ_*t*_ represents the depreciation rater. As illustrated in Figure 1, the intersection of the benefit curve and the cost curve for health determines the optimal demand for health, denoted as ${H_{t}}^{*}$. We simplify health investment as the utilization of medical services. In this case, if the cost of utilizing medical services decreases, it will only reduce the marginal cost of health investment (π_t − 1_). The benefits curve, derived from $\gamma_{t}=\frac{W_{t}G_{t}}{\pi_{t-1}}$ and $\alpha_{t}=\frac{G_{t}\left[\left(\frac{U_{ht}}{m}\right){\left(1+r\right)}^{t}\right]}{\pi_{t-1}}$, will shift to the right, leading to an increase in the consumer's optimal demand ${H_{t}}^{*}$ for health. The increase in health demand results in higher utilization of medical services by the consumer.

**Figure 2:**
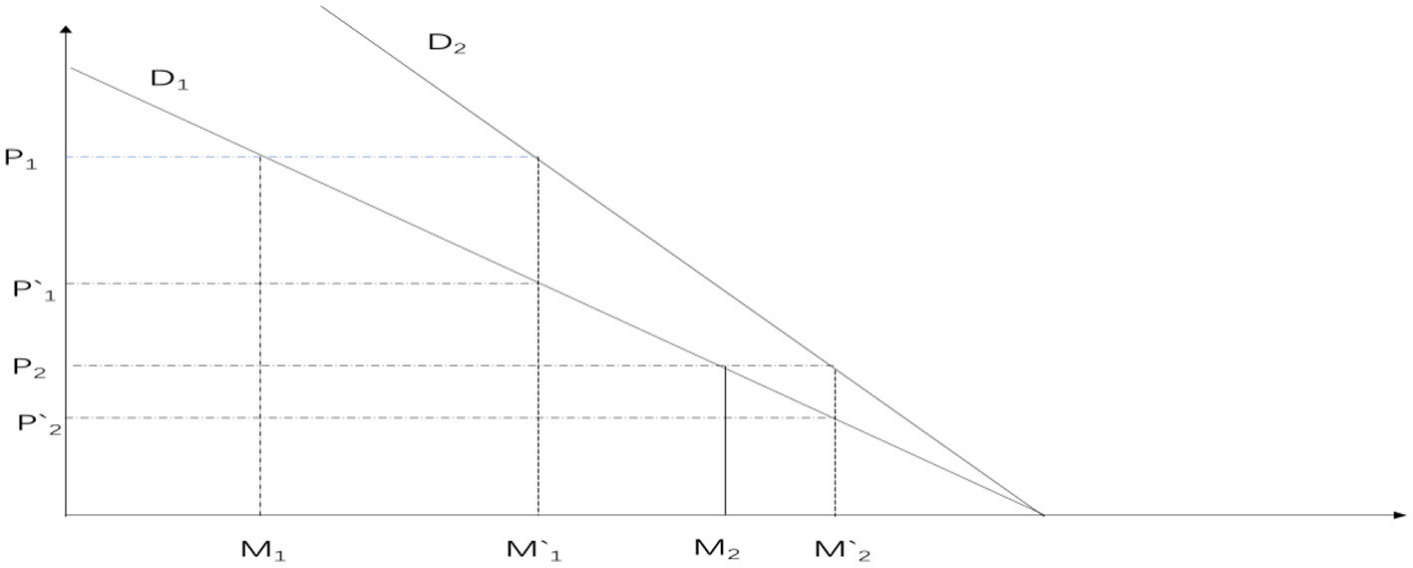
The impact of health insurance system on consumer medical demand curve.

The promotion of health through increased utilization of healthcare services is unquestionable (7, 8, 10). As deduced from the previous Grossman model, healthcare services are considered normal goods. When the price of healthcare services (P) decreases, it implies a reduction in the marginal cost of health investment, thereby increasing the marginal return on health. Hence, consumers' demand for health and medical services will increase. Under the original coverage of the New Rural Cooperative Medical Scheme (NRCMS), the slope of the consumer's medical demand curve is negative, as represented by the D_1_ line in Figure 2. Assuming that the integration of health insurance reduces the price consumers face when seeking medical care from P_1_ under the NRCMS to P‘_1_ under the integrated urban and rural resident health insurance, consumers' demand for medical services will increase from M_1_ to M‘_1_. This change can be illustrated in Figure 2 by the shift from the original D_1_ to the new D_2_ curve. In other words, the improvement in health insurance benefits, leading to a decrease in the actual price consumers pay for medical services during treatment, will naturally increase the demand for medical services, thereby promoting health. From a practical perspective, the integration of urban and rural resident health insurance can improve the health status of rural residents, after the integration of urban and rural resident health insurance, the reimbursement rate for rural residents' medical expenses will increase. Taking the example of the inpatient reimbursement rate, which undergoes the most significant change, the reimbursement rate for hospitalization expenses within the payment policy scope of New Rural Cooperative Medical Scheme (NRCMS) is ~56.6%, while the reimbursement rate for hospitalization expenses within the payment policy scope of urban and rural resident health insurance is about 69.3% (11, 12).

Furthermore, during the transformation from New Rural Cooperative Medical Scheme (NRCMS) to the Urban and Rural Resident health insurance (URRMI), this process may have heterogeneous effects on the health of middle-aged and older people individuals with varying health conditions. Middle-aged and older people individuals, due to the aging process, often experience a significant decline in their health compared to when they were younger. Moreover, because of differences in long-term health accumulation, there are substantial variations in health among this population. Individuals with different health statuses tend to have different types of illnesses, and the medical expenses incurred when seeking healthcare also vary. Relatively speaking, individuals with poorer health are more likely to develop severe illnesses, resulting in much higher medical expenses compared to those with milder health conditions. Furthermore, health insurance reimbursement rates do not decrease as medical prices increase. On the contrary, China's existing health insurance system often includes supplementary insurance for severe illnesses for individuals with significant healthcare needs. Urban and rural catastrophic illness insurance is designed to reimburse urban and rural residents for the substantial medical expenses incurred due to severe illnesses. Its primary goal is to prevent urban and rural residents from facing catastrophic medical expenditures. The insurance system establishes payment ratios segmented based on the level of medical expenses. In principle, as medical expenses increase, the payment ratio also increases, effectively minimizing the personal financial burden of medical costs. This, in turn, further elevates the reimbursement rates for individuals with severe illnesses, alleviating concerns about the high cost of healthcare and encouraging them to seek medical treatment.

Moreover, the medical services required by patients with severe illnesses are usually much more expensive than those for individuals with milder conditions. As depicted in Figure 2, the medical price for patients with severe illnesses is represented as P1, while for patients with milder conditions, it is P2. After the integration of health insurance, the reimbursement rates become P‘_1_ and P‘_2_, respectively. However, the change in optimal medical services for patients with severe illnesses (M_1_‘-M_1_) is significantly greater than that for patients with milder conditions (M_2_‘-M_2_). This implies that the impact on the health of patients with severe illnesses is greater, aligning with the primary function of health insurance to “protect against major illnesses.” Based on this, we propose the hypothesis:

**Hypothesis:** The integration of urban and rural resident health insurance can promote the health of rural middle-aged and older people individuals, with a greater impact on those with lower health levels.

## 3 Data source and research methods

### 3.1 Data source

The data used in this study is sourced from the China Health and Retirement Longitudinal Study (CHARLS). CHARLS is a large interdisciplinary survey project conducted by the National School of Development at Peking University, in collaboration with the China Social Science Survey Center at Peking University and the Beijing University Youth League Committee. It primarily focuses on individuals aged 45 and above. The baseline survey of CHARLS in China was conducted in 2011, with follow-up surveys conducted every 2–3 years. Considering that the integration of rural and urban resident health insurance systems took place gradually in different regions, the large-scale integration of the New Rural Cooperative Medical Scheme (NRCMS) and the Urban Resident Basic health insurance (URBMI) into the Urban and Rural Resident health insurance (URRMI) occurred in 2016 following the issuance of the State Council's “Opinions on Integrating the Basic health insurance Systems for Urban and Rural Residents.” The policy impact primarily occurred between 2015 and 2018. Based on this, this study utilizes the most recent data from the 2015 and 2018 waves of CHARLS.

### 3.2 Variable selection

#### 3.2.1 Health

Health is commonly defined as a state of complete physical, mental, and social wellbeing and not merely the absence of disease or infirmity. Health is the foundation of human survival and development, serving as not only a prerequisite for survival but also as the basis for realizing individual potential and pursuing happiness. To address the limitations of traditional health measurement indicators, many scholars in recent years have used frailty indices to reflect the health and aging status of middle-aged and older people individuals (13, 14). The frailty index (FI), also known as the health deficit index, refers to the proportion of health measurement indicators for which an individual's value is considered unhealthy. It has a range of values from 0 to 1, with a higher frailty index indicating poorer health. This study comprises the following six modules:

(1) Self-rated health (HEALTH): How do you rate your health? Define it as 0.2 for “very good,” 0.4 for “good,” 0.6 for “fair,” 0.8 for “bad,” and 1 for “very bad.”
(2) Instrumental Activities of Daily Living (IADL): Do you have difficulty with “managing money, shopping, cooking, making phone calls, and housekeeping?” If you have difficulty, define it as 1, otherwise as 0.
(3) Activities of Daily Living (ADL): Do you have difficulty with “bathing, getting out of bed, using the toilet, eating, dressing, and making decisions?” If you have difficulty, define it as 1, otherwise as 0.
(4) Functional Limitations: Do you have difficulty with activities such as “walking 100 m, climbing stairs, reaching up, getting up from a chair, bending or kneeling or squatting, picking up a coin, carrying a 10-kg weight?” If you have difficulty, define it as 1, otherwise as 0.
(5) Mini-Mental State Examination (MMSE): Can you clearly answer, “What year is it? What month is this? What is the date today? What season is it now? What day of the week is today? How is your memory right now? Can you draw the picture you see? And what is your level of depression?,”^1^ Memory, similar to self-rated health, is defined as follows: “very good” is defined as 0.2, “good” as 0.4, “fair” as 0.6, “bad” as 0.8, and “very bad” as 1. All other responses are correctly defined as 0, and any other answers are defined as 1.
(6) Chronic diseases, based on whether one has them or not, including conditions like hypertension, hyperlipidemia, hyperglycemia, malignant tumors, chronic lung diseases, liver diseases, heart diseases, stroke, kidney diseases, gastrointestinal diseases, emotional and mental issues, memory-related diseases, rheumatism, and asthma, are defined as 0 (absent) or 1 (present).

The above-mentioned six modules collectively involve 41 health variables, with specific formulas as follows:


(5) $$\begin{array}{l} FI=\frac{\overset{n}{\underset{k=1}{\sum }}d_{i}}{n} \end{array}$$

In the above formulas, FI represents the frailty index, with *n* = 41, and *d*_*i*_ = 1 indicates that the i-th health variable is in a compromised health state; otherwise, *d*_*i*_ = 0. The frailty index is a continuous variable ranging from 0 to 1, where a higher frailty index indicates a poorer health condition of the surveyed individuals.

#### 3.2.2 The integration of urban and rural residents' health insurance

First, individuals who participated continuously in both 2015 and 2018 were selected. This includes individuals who were enrolled in the New Rural Cooperative Medical Scheme (NCMS) in 2015 and remained enrolled in the NCMS in 2018, as well as individuals who were enrolled in the NCMS in 2015 and switched to the Urban and Rural Resident Basic health insurance (URRBMI) in 2018. Individuals' participation in the integrated Urban and Rural Resident Basic health insurance (URRBMI) can be identified based on their responses regarding the type of health insurance they had (6, 15–17). This information can be used to determine whether they experienced the integration of rural and urban resident health insurance schemes. To mitigate the possibility of rural middle-aged and older people individuals being uninformed about this policy due to information gaps, a household-based approach was adopted to ascertain their insurance participation status. If at least one member within their household had undergone the integration of Urban and Rural Resident Basic health insurance (URRBMI), then the rural middle-aged or older people individual was considered to have experienced the integration of URRBMI. Furthermore, we conducted several exclusions in the dataset. Firstly, we removed individuals who were not enrolled in any insurance scheme, those enrolled in the Urban Employee health insurance program, cases of duplicated insurance enrollment, individuals with commercial health insurance coverage, and participants who were not locally enrolled. We also excluded individuals who were already enrolled in the Urban and Rural Resident Basic health insurance (URRBMI) before 2015. As the primary focus of this study is rural middle-aged and older people individuals, we excluded individuals not residing in rural areas. Finally, we excluded data points where insurance status could not be determined and those with missing variable information. This indicator takes a value of 1 for individuals who experienced the integration of rural and urban resident basic health insurance (URRBMI), and a value of 0 for those who remained under the New Cooperative Medical Scheme (NCMS) coverage.

#### 3.2.3 Control variables

This study selects control variables from five main aspects:

1. Individual characteristics, including gender (female = 1, male = 0), age, marital status (married or cohabiting = 1, unmarried, divorced, widowed = 0), income (household per capita annual income), years of education (no education or incomplete primary school = 0; primary school = 6; junior high school = 9; high school or vocational school = 12; college degree = 15; bachelor's degree = 16; master's degree = 19; doctorate = 23), number of children (total number of surviving biological and stepchildren), etc. Health behaviors: This encompasses smoking status (smoking = 1; non-smoking = 0), physical exercise (exercise = 1; no exercise = 0), alcohol consumption (drinking = 1; not drinking = 0), and others.
2. Health behaviors: This encompasses smoking status (smoking = 1; non-smoking = 0), physical exercise (exercise = 1; no exercise = 0), and alcohol consumption (drinking = 1; not drinking = 0).
3. Household hygiene environment: The cleanliness of the household is represented by a cleanliness scale (1 = very clean; 2 = quite clean; 3 = clean; 4 = average; 5 = not clean).
4. Quality of medical services and healthcare costs: This is measured by satisfaction with the level of services provided by medical institutions (1 = very satisfied; 2 = satisfied; 3 = neutral; 4 = dissatisfied; 5 = very dissatisfied).
5. Regional fixed effects: Given the varying levels of economic development across different regions, this study controls for regional fixed effects at the provincial level (7, 18, 19). The specific details of each variable can be found in Table 1.

**Table 1:** Descriptive statistical analysis of the impact of the integration of urban and rural residents' health insurance on health.

**Variable**	**Full sample**		**Experimental group**		**Control group**	
	**Mean**	**Standard deviation**	**Mean**	**Standard deviation**	**Mean**	**Standard deviation**
URRBMI	0.0930	0.2905	0.5814	0.4935	0	0
Frailty index	0.1656	0.1098	0.1553	0.1073	0.1675	0.1102
Age	61.8287	9.3020	61.9919	9.4282	61.7976	9.2781
Marital status	0.8427	0.3641	0.8582	0.3489	0.8397	0.3669
Gender	0.5207	0.4996	0.5114	0.5000	0.5225	0.4995
Logarithm of per capita income	7.0420	3.0365	7.3565	2.9891	6.9821	3.0420
Smoking	0.2898	0.4537	0.2694	0.4438	0.2937	0.4555
Alcohol consumption	0.3365	0.4725	0.3329	0.4714	0.3371	0.4728
Physical exercise	0.9234	0.2660	0.9068	0.2907	0.9266	0.2610
Medical satisfaction	2.6387	1.1055	2.5726	1.0554	2.6513	1.1144
Household cleanliness	3.0485	1.0731	3.0209	1.0496	3.0538	1.0776
Number of children	3.0334	1.5762	2.8629	1.3730	3.0659	1.6101

#### 3.2.4 Variable descriptive statistics

Table 1 presents the results of the descriptive statistical analysis. In the preliminary examination, it appears that there are not substantial differences between the experimental group and the control group across various variables. Nonetheless, an empirical research analysis is required to assess the impact of the integration of urban and rural residents' health insurance on rural older people individuals.

### 3.3 Research method

#### 3.3.1 Difference-in-Differences (DID) methodology

Based on the basic steps outlined in the DID model, construct two dummy variables: First, construct dummy variables for the “experimental group” and “control group.” “The experimental group” consists of residents who were covered by the New Rural Cooperative Medical Scheme (NRCMS) in the base year of 2015 and transitioned to the integrated Urban and Rural Residents Basic health insurance (URRBMI) in 2018. The “control group” includes individuals who were covered by NRCMS in both 2015 and 2018. Second, create policy timing dummy variables. By comparing the differences in the health status between the “experimental and control groups,” analyze the impact of the integration of urban and rural residents' health insurance on rural middle-aged and older people individuals. Based on the above analysis, the specific regression model using the Difference-in-Differences (DID) method is as follows:


(6) $$\begin{array}{l} Y_{it}=\beta_{0}+\beta_{1}DID_{it}+\beta_{2}Treat_{i}+\beta_{3}Time_{t}+\delta X_{it}+\epsilon_{it} \end{array}$$

(7) $$\begin{array}{l} DID_{it}=Treat_{i}\times Time_{t} \end{array}$$

Understood. In the model notation, i represents individual or resident, t represents the time period, and *Y*_*it*_ represents the health indicator of individual i in time period t.*DID*_*it*_ is the core explanatory variable. *Treat*_*i*_ is a group dummy variable, where *Treat*_*i*_ = 1 if i belongs to the experimental group; otherwise, if i belongs to the control group, *Treat*_*i*_ = 0. *Time*_*t*_ is a time-period dummy variable, where *Time*_*t*_=1 if i is in the year 2018; otherwise, if i is not in 2018, ϵ_*it*_ represents the disturbance term. Through the above screening process, a balanced panel dataset was formed with 1,481 observations in the experimental group and 7,774 observations in the control group across two periods.

#### 3.3.2 Quantile Difference-in-Differences (Q-DID)

The linear assumptions of the OLS model are likely to mask the effects on older people individuals with different levels of vulnerability. Quantile evaluation methods can comprehensively assess changes in various parameters at different quantile points of the dependent variable (20). Therefore, this study employs the quantile difference-in-differences regression method to investigate the health effects at different quantile points. Setting as follows:


(8) $$\begin{array}{l} \tau \left(Y_{it}\right)=\beta_{0}+\beta_{1}DID_{it}+\beta_{2}Treat_{i}+\beta_{3}Time_{t}+\delta X_{it}+\epsilon_{it} \end{array}$$

τ(*Y*_*it*_) represents the quantile of individual i's health status at time t. The coefficients at different quantiles indicate the impact of urban-rural resident health insurance integration on the health status at that quantile.

## 4 Baseline regression analysis of the impact of urban-rural resident health insurance integration on health

### 4.1 Parallel trends test

**Figure 3:**
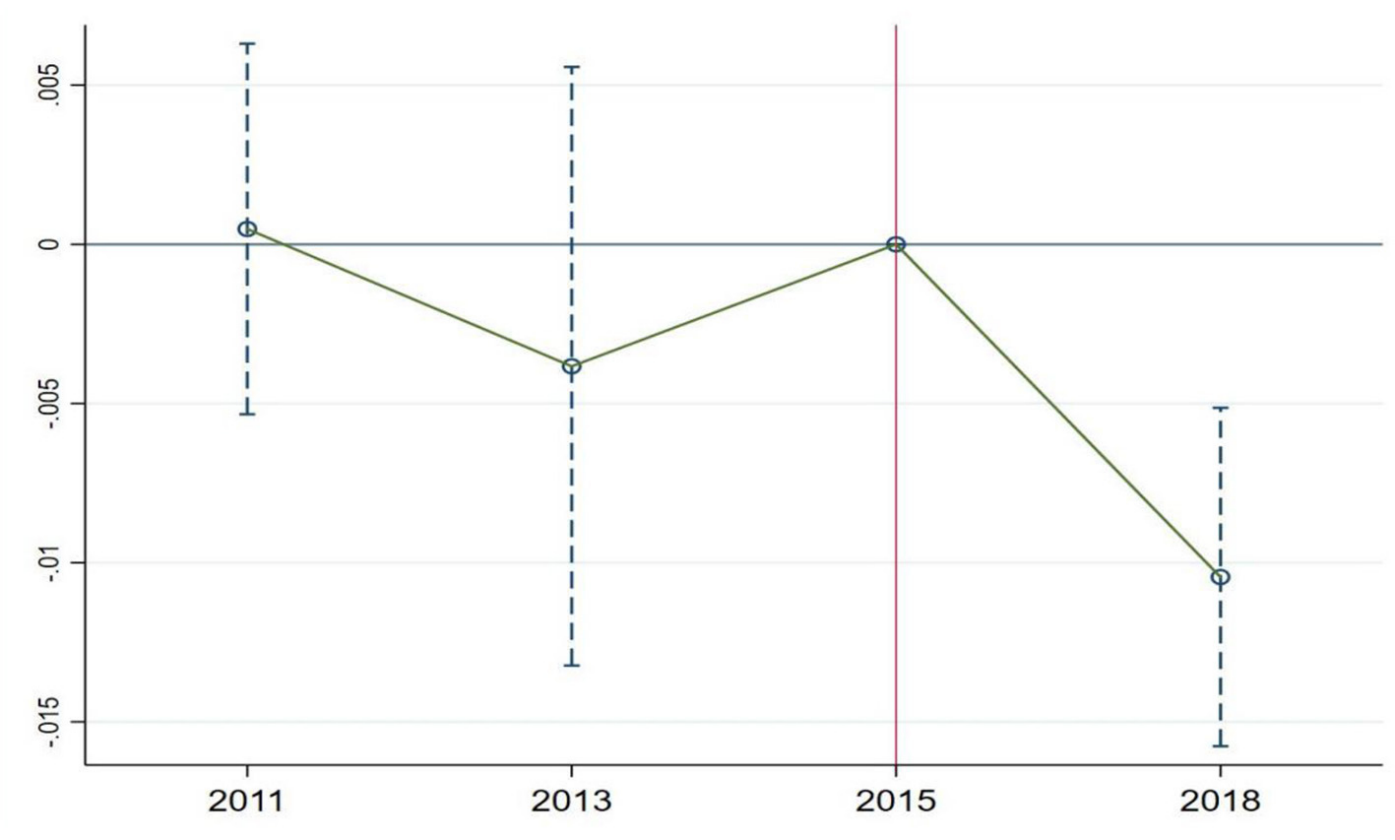
Parallel trends test for health.

The double difference method correctly identifies causal effects under the assumption that the experimental group and the control group have parallel trends before the policy shock. Researchers typically indirectly test the parallel trends assumption by examining whether the pre-treatment trends of the experimental and control groups are the same. This study follows the approach used by Huang et al. to avoid issues of collinearity (21). The base period is set as the year immediately prior to the policy implementation (2015) when the policy took effect (2016). As shown in Figure 3, the coefficients for 2011 and 2013 are not significantly different from 0, indicating that there were no significant differences between the experimental group and the control group in 2011 and 2013 compared to 2015. Therefore, it can be assumed that the pre-treatment parallel trends assumption is satisfied. However, due to the availability of only four waves of CHARLS data, with only three periods before the policy change, it can be challenging to precisely assess parallel trends. To ensure the robustness of the results, this study will further employ robustness analysis methods such as instrumental variable estimation, PSM-DID, and placebo tests in subsequent sections.

### 4.2 Baseline regression

As shown in Table 2, to minimize the impact of omitted variables, Models (1–3) report the regression results under the difference-in-differences (DID) approach without controlling for covariates and province fixed effects, with covariates but without province fixed effects, and with all covariates and regional fixed effects, respectively. Without adding covariates and regional fixed effects, the effect of urban-rural resident health insurance integration on the frailty index is −0.0176. When adding covariates but not province fixed effects, the effect is −0.0200. With all covariates and regional fixed effects included, the effect is −0.0198. The coefficient of the impact remains relatively stable, indicating the robustness of the results. Moreover, whether or not additional control variables and regional fixed effects are included, the effect of urban-rural resident health insurance integration on the frailty index remains statistically significant at the 1% level. Hypothesis 2 has been validated. Although in the short term, the urban-rural resident health insurance integration policy has shown a relatively limited improvement in residents' health, it's important to note that health is a long-term accumulation of capital (1). The impact of urban-rural resident health insurance integration on health is expected to become more significant as time progresses.

**Table 2:** Baseline regression of the impact of urban-rural resident health insurance integration on health.

**Variables**	**Frailty index**		
	**(1)**	**(2)**	**(3)**
DID	−0.0178^***^	−0.0200^***^	−0.0198^***^
	(0.0047)	(0.0044)	(0.0043)
Treat	−0.0025	0.0006	0.0062
	(0.0049)	(0.0045)	(0.0044)
Time	0.0084^***^	−0.0036^*^	−0.0045^**^
	(0.0017)	(0.0021)	(0.0021)
Age		0.0026^***^	0.0027^***^
		(0.0002)	(0.0002)
Gender		0.0278^***^	0.0280^***^
		(0.0033)	(0.0032)
Per capita income		−0.0018^***^	−0.0018^***^
		(0.0004)	(0.0004)
Marital status		−0.0027	−0.0036
		(0.0032)	(0.0032)
Exercise		−0.0507^***^	−0.0523^***^
		(0.0053)	(0.0051)
Smoking		0.0042	0.0053^*^
		(0.0029)	(0.0029)
Education years		−0.0049^***^	−0.0050^***^
		(0.0003)	(0.0003)
Alcohol consumption		0.0127^***^	0.0122^***^
		(0.0026)	(0.0025)
Household cleanliness		0.0102^***^	0.0095^***^
		(0.0010)	(0.0010)
Medical satisfaction		0.0096^***^	0.0090^***^
		(0.0010)	(0.0010)
Number of children		0.00411^***^	0.0037^***^
		(0.0009)	(0.0010)
Constant	0.1625^***^	0.0120	0.0212
	(0.0018)	(0.0127)	(0.0133)
Regional effects	No	No	Yes
*R* ^2^	0.0032	0.2231	0.2606
Sample size	9255	9255	9255

Standard errors in parentheses, ^***^,^**^, and ^*^ correspond to the 1%, 5%, and 10% significance levels, respectively.

In terms of control variables, as individuals age, their physical condition tends to gradually decline, leading to an increase in frailty. Being married, on the other hand, provides more companionship and care. Having a spouse is advantageous for improving health. Income and educational level are directly related to health awareness and health investments. Higher income and educational attainment are associated with better health. Unhealthy habits, such as smoking and alcohol consumption, have a significant negative impact on health, while regular physical exercise can improve health. Improvements in the quality of healthcare services are beneficial for receiving better medical care, which can lead to improved health. Having more children can increase the financial burden on parents, potentially leading to worse health outcomes.

Furthermore, this study conducted regression analyses on different health sub-module indicators. The results showed that whether it's self-rated health, daily activity ability, instrumental activity ability, physical function limitations, chronic disease status, or mental health status, rural older people residents' health significantly improved due to the integration of urban and rural resident health insurance. Among them, self-rated health and mental health are significant at the 1% level, while daily activity ability, instrumental activity ability, and physical function limitations are significant at the 5% level. Chronic disease status is significant at the 10% level (Table 3).

**Table 3:** Regression analysis of the impact of urban and rural residents' health insurance integration on different health indicators.

**Variables**	**Self-rated health**	**Daily abilities**	**Tool abilities**	**Functional limitations**	**Chronic diseases**	**Mental status**
DID	−0.0166^*^	−0.1225^***^	−0.1212^***^	−0.1427^**^	−0.1295^*^	−0.2706^***^
	(0.0086)	(0.0428)	(0.0440)	(0.0644)	(0.0729)	(0.0722)
Treat	0.0033	0.0321	0.0121	0.0262	0.0761	0.1070^*^
	(0.0085)	(0.0415)	(0.0385)	(0.0657)	(0.0660)	(0.0620)
Time	0.0285^***^	0.0473^**^	0.1274^***^	0.2725^***^	−0.9155^***^	0.2139^***^
	(0.0042)	(0.0204)	(0.0216)	(0.0331)	(0.0329)	(0.0321)
Control variables	Yes	Yes	Yes	Yes	Yes	Yes
Regional effects	Yes	Yes	Yes	Yes	Yes	Yes
*R* ^2^	0.1072	0.0993	0.1224	0.2050	0.1669	0.1989
Sample size	9255	9255	9255	9255	9255	9255

Standard errors in parentheses, ^***^ denotes significance at the 1% level, ^**^ at the 5% level, and ^*^ at the 10% level.

On one hand, through regression analysis of different health sub-module indicators, it has been demonstrated that the baseline regression is robust, confirming that the integration of urban and rural residents' health insurance has indeed improved the health status of rural older people individuals. On the other hand, it has also been discovered that the integration of urban and rural residents' health insurance has led to a comprehensive improvement in the health status of rural older people individuals. This improvement is evident in various aspects, including subjective health as reflected in self-rated health, as well as objective physiological health conditions reflected through daily activity capacity, instrumental activity capacity, functional limitations, chronic disease status, and mental health as reflected in psychological and emotional wellbeing. This can be attributed to the fact that the integration of urban and rural residents' health insurance has not only significantly increased the reimbursement rates for medical expenses among rural older people individuals but has also expanded the scope of health insurance coverage and the number of designated hospitals. It has comprehensively safeguarded the healthcare rights and interests of rural older people individuals, leading to significant improvements in the treatment of various diseases and an evident enhancement in their overall health status.

The Chinese basic health insurance system focuses on providing significant coverage for major illnesses, while the coverage for basic diseases is not very high. From this perspective, health insurance is expected to have a greater marginal effect on improving the health of people with serious illnesses, who have lower health levels. In other words, the impact of the integration of urban and rural residents' health insurance on the health of rural older people people should also be non-linear. To test this hypothesis, this study used quantile double-difference regression. The results, as shown in Table 4, reveal that in the quantile double-difference regression, the integration of urban and rural residents' health insurance has a negative impact on the frailty index. Since the frailty index is a negative indicator, this indicates that the integration of urban and rural residents' health insurance significantly improves the health of rural older people individuals. More importantly, this health impact varies significantly at different quantiles of the frailty index.

**Table 4:** Quantile double difference regression of the impact of urban-rural resident health insurance integration on health.

**Variables**	**Q10**	**Q25**	**Q50**	**Q75**	**Q90**
DID	−0.0108^**^	−0.0127^***^	−0.0130^***^	−0.0218^***^	−0.0404^***^
	(0.0048)	(0.0045)	(0.0046)	(0.0071)	(0.0139)
Treat	0.0024	0.0047	0.0035	0.0043	0.0145
	(0.0033)	(0.0032)	(0.0054)	(0.0066)	(0.0099)
Time	0.0008	0.0012	−0.0035	−0.0047	−0.0080
	(0.0019)	(0.0027)	(0.002)	(0.0034)	(0.0053)
Age	0.0011^***^	0.0018^***^	0.0026^***^	0.0035^***^	0.0044^***^
	(0.0001)	(0.0002)	(0.0001)	(0.0002)	(0.0004)
Gender	0.0159^***^	0.0255^***^	0.0291^***^	0.0334^***^	0.0363^***^
	(0.0016)	(0.0025)	(0.0029)	(0.0035)	(0.0064)
Per capita income	−0.0008^***^	−0.0013^***^	−0.0013^***^	−0.0020^***^	−0.0032^***^
	(0.0002)	(0.0002)	(0.0005)	(0.0008)	(0.0011)
Marital status	−0.0056	−0.0064^**^	−0.0055^*^	−0.0059	0.0045
	(0.0034)	(0.0030)	(0.0033)	(0.0042)	(0.0064)
Exercise	−0.0150^***^	−0.0211^***^	−0.0454^***^	−0.0742^***^	−0.0922^***^
	(0.0046)	(0.0044)	(0.0052)	(0.0073)	(0.0092)
Smoking	0.0022	0.0018	0.0014	0.0065^**^	0.0104^*^
	(0.0021)	(0.0024)	(0.0021)	(0.0032)	(0.0057)
Years of education	−0.0022^***^	−0.0029^***^	−0.0043^***^	−0.0057^***^	−0.0083^***^
	(0.0002)	(0.0002)	(0.0003)	(0.0004)	(0.0004)
Alcohol consumption	0.0011	0.0018	0.0082^***^	0.0143^***^	0.0238^***^
	(0.0012)	(0.0023)	(0.0026)	(0.0037)	(0.0037)
Household cleanliness	0.0044^***^	0.0060^***^	0.0068^***^	0.0097^***^	0.0168^***^
	(0.0006)	(0.0008)	(0.0008)	(0.0013)	(0.0016)
Medical satisfaction	0.0049^***^	0.0061^***^	0.0070^***^	0.0098^***^	0.0130^***^
	(0.0008)	(0.0008)	(0.0009)	(0.0013)	(0.0020)
Number of children	0.0030^***^	0.0027^***^	0.0032^***^	0.0041^***^	0.0038^*^
	(0.0008)	(0.0008)	(0.0008)	(0.0013)	(0.0022)
Constant term	−0.0074	−0.0132	0.0056	0.0404^**^	0.0554^**^
	(0.0103)	(0.0139)	(0.0111)	(0.0176)	(0.0242)
Regional effects	Yes	Yes	Yes	Yes	Yes
Sample size	9,255				

Standard errors in parentheses. ^***^, ^**^, and ^*^ denote significance levels of 1%, 5%, and 10%, respectively.

**Figure 4:**
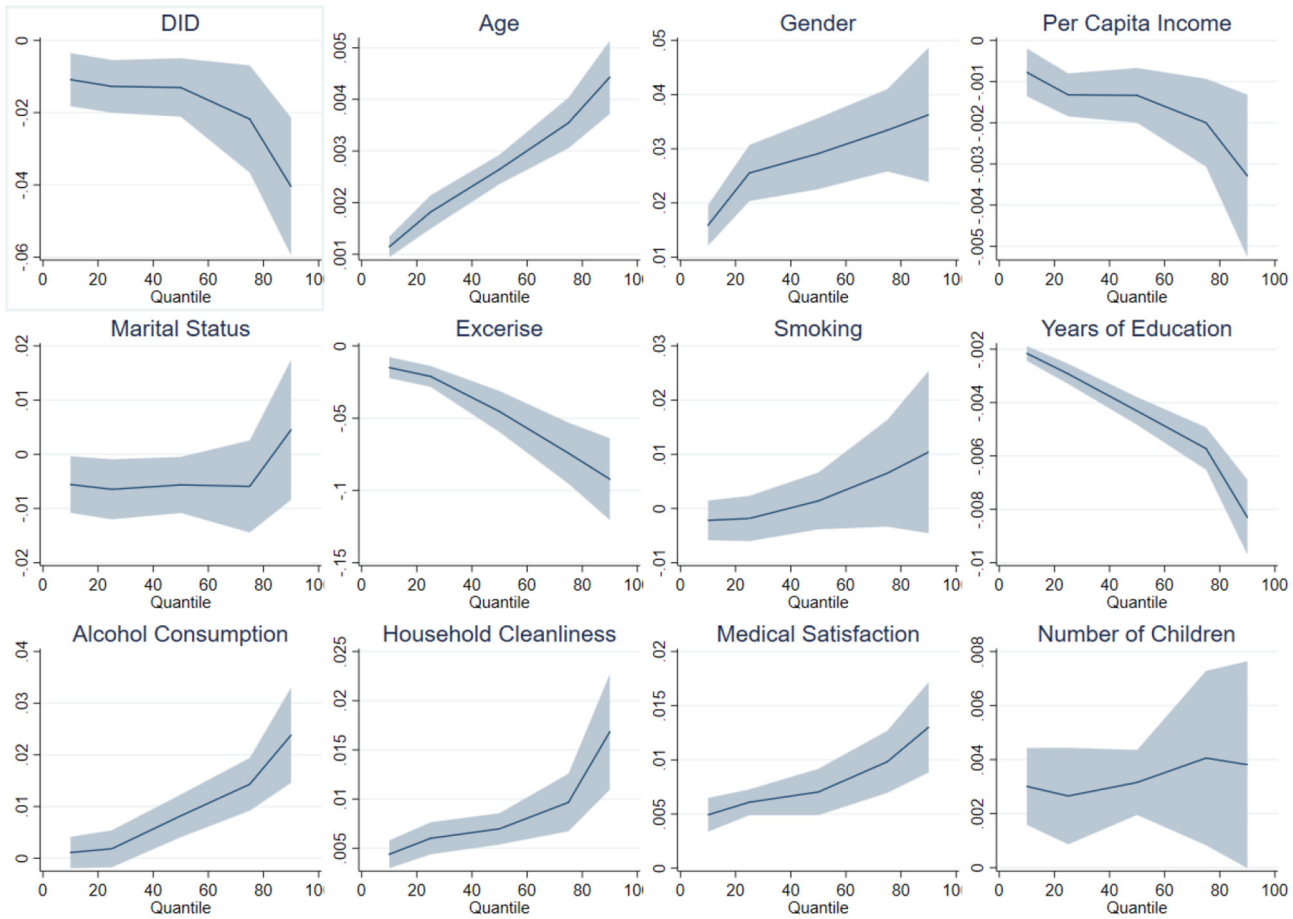
Quantile coefficient effects of urban and rural resident health insurance integration on health.

Assuming that the health impact is linear, combined with the previous OLS regression, it is found that the average effect of integrating urban and rural residents' health insurance on the frailty index is a reduction of 0.0198. However, in the quantile double difference regression, at the 10, 25, 50, 75, and 90th percentiles, the integration of urban and rural residents' health insurance led to reductions in the frailty index of 0.0108, 0.0127, 0.0130, 0.0218, and 0.0404, respectively, and these effects were significant at least at the 5% level (Table 4). From the quantile perspective, the impact of the integration of urban and rural residents' health insurance on the frailty index was relatively consistent up to the 50th percentile. However, beyond the 50th percentile, the impact on the frailty index gradually increased, with the most significant effect observed at the 90th percentile, indicating the strongest promotion of health. This study has plotted a trend chart of quantile regression coefficients to provide a more visual representation of this effect (Figure 4).

### 4.3 Path analysis of the impact of urban and rural resident health insurance integration on health

Health insurance can improve residents' health by reducing the financial burden of medical care, increasing the utilization of medical services, and enhancing overall health outcomes. Previous literature has analyzed the pathways through which health insurance affects health, providing valuable insights for the analysis in this study (7, 8, 10, 22–25). This study primarily examines why the integration of urban and rural residents' health insurance has a greater impact on improving the health of rural older people individuals with lower health status. The integration of urban and rural residents' health insurance primarily affects the health status of residents by reducing the financial burden of healthcare. It directly lowers the cost of medical expenses and enhances the utilization of medical services by residents. However, considering the significant amount of missing data in the outpatient and inpatient medical service utilization in the CHARLS dataset, this study, following the approach of Chang et al., chooses healthcare expenditure (annual medical consumption as a proportion of annual total consumption) as a mediating variable in the causal pathway (26). Building on this, the study further investigates the impact of the integration of urban and rural residents' health insurance on the mediating variable, as shown in Table 5. It is evident that the integration of urban and rural residents' health insurance significantly reduces the healthcare expenditure burden on rural older people individuals.

**Table 5:** The impact of the integration of urban and rural residents' health insurance on healthcare expenditure burden.

**Variables**	**Healthcare expenditure burden**
DID	−0.0381^**^
	(0.0182)
Treat	0.0263^*^
	(0.0153)
Time	0.0795^***^
	(0.0078)
Regional effects	Yes
Control variables	Yes
Sample size	8,934

Standard errors in parentheses, ^***^ denotes significance at the 1% level, ^**^ at the 5% level, and ^*^ at the 10% level.

Following this, this study incorporates medical burden as a core variable into the quantile double difference method, as shown in Table 6. The effect of medical burden on the frailty index is significantly positive, and the DID regression coefficient remains significant at this point, indicating that the integration of urban and rural residents' health insurance does improve health by reducing medical burden. Furthermore, this study examined the marginal effects of medical burden at different quantile points. The regression coefficients for medical burden and DID remained significant at various quantile points, with their impact increasing as the quantile points increased. Based on the above analysis, this study concludes that the integration of urban and rural resident health insurance reduces the medical burden on patients, relaxes their budget constraints, and has a greater impact on patients with severe illnesses. It encourages more utilization of medical services and investment in healthcare, thereby improving the health of individuals with lower health levels. This further emphasizes the role of health insurance in providing protection against major illnesses.

**Table 6:** Pathway analysis of the impact of integrated urban and rural resident health insurance on health.

	**Full sample**	**Q10**	**Q20**	**Q50**	**Q75**	**Q90**
Medical burden	0.0427^***^	0.0179^***^	0.0228^***^	0.0405^***^	0.0552^***^	0.0756^***^
	(0.0036)	(0.0025)	(0.0027)	(0.0034)	(0.0045)	(0.0087)
DID	−0.0190^***^	−0.0075^*^	−0.0093^**^	−0.0119^**^	−0.0210^***^	−0.0273^***^
	(0.0052)	(0.0040)	(0.0042)	(0.0052)	(0.0063)	(0.0086)
Control variables	Yes	Yes	Yes	Yes	Yes	Yes
Regional effects	Yes	Yes	Yes	Yes	Yes	Yes
Sample size	8,934					

Standard errors in parentheses, ^***^, ^**^, ^*^ represent significance levels of 1%, 5%, and 10%, respectively.

### 4.4 Heterogeneity analysis of the impact of urban and rural residents' health insurance integration on health

As shown in Table 7, Panel A divides the sample into two groups based on age: the middle-aged group (Age < 60 years) and the older people group (Age ≥ 60 years). In different groups of older people and middle-aged individuals, the coefficient of the DID estimate is significantly larger for the older people group than for the middle-aged group. This could be due to the lower health status of older people individuals, where the integrated urban-rural resident health insurance policy has a greater impact on those with lower health status, resulting in greater benefits for the older people group. Overall, the health performance of the integrated urban-rural resident health insurance policy is more favorable for the older people, and its impact is most pronounced among the older people with the poorest health status. Furthermore, looking at different quantiles, the impact of integrated urban-rural resident health insurance on the vulnerability index increases with higher quantiles, and its impact on the older people is greater than on the middle-aged group at each quantile point.

**Table 7:** Heterogeneity analysis of the impact of urban-rural resident health insurance integration on health.

	**Full sample**	**Q10**	**Q20**	**Q50**	**Q75**	**Q90**
Middle-aged	−0.0161^***^	−0.0051	−0.0081^*^	−0.0153^**^	−0.0261^**^	−0.0228
*N* = 4,666	(0.0059)	(0.0049)	(0.0043)	(0.0069)	(0.1262)	(0.0207)
Older people	−0.0198^***^	−0.0183^**^	−0.0112^**^	−0.0061	−0.0205^**^	−0.0466^**^
*N* = 6,136	(0.0064)	(0.0072)	(0.0056)	(0.0066)	(0.0089)	(0.0232)
Eastern region	−0.0180^***^	−0.0080	−0.0132^**^	−0.0106^*^	−0.0205^**^	−0.0249
*N* = 3,556	(0.0056)	(0.0065)	(0.0052)	(0.0060)	(0.0081)	(0.0153)
Central region	−0.0205^**^	−0.0012	−0.0102	−0.0160	−0.0376^*^	−0.0480
*N* = 3,180	(0.0089)	(0.0070)	(0.0120)	(0.0114)	(0.208)	(0.3048)
Western region	−0.0232^**^	−0.0154^*^	−0.0069	−0.0096	−0.0146	−0.0223
*N* = 4,066	(0.0096)	(0.0086)	(0.0086)	(0.0106)	(0.0142)	(0.0197)

Standard errors in parentheses, ^***^ denotes significance at the 1% level, ^**^ denotes significance at the 5% level, and ^*^ denotes significance at the 10% level. All models control for regional effects and control variables.

As shown in Table 7, Panel B, the groups are divided based on regions, including the Western region, Central region, and Eastern region. The impact of the urban-rural resident health insurance integration policy on the health of residents decreases in the following order: Western region, Central region, and Eastern region. On one hand, the decreasing impact of the urban-rural resident health insurance integration policy on the health of residents in Eastern, Central, and Western regions is due to the declining health levels in these regions, and the policy has a larger marginal effect on residents with lower health status. On the other hand, after the 13th Five-Year Plan, the Chinese government further strengthened medical resource support in Western and Central regions through initiatives like East-West regional pairing support and the promotion of county-level medical consortiums in these regions. This increased support for medical resources has improved the quality of healthcare delivery and contributed to a more balanced distribution of healthcare resources in these regions. It has gradually reduced regional healthcare imbalances, promoted equitable basic public health services, and alleviated issues related to regional development disparities. In different regions, the quantile regression results are generally consistent with the baseline regression. As the quantile levels increase, the impact becomes larger.

### 4.5 The robustness examination of the impact of integrated urban and rural resident health insurance on health

#### 4.5.1 Instrumental variable test

Due to variations in the implementation of urban-rural resident health insurance integration policies in different regions, as well as differences in public awareness of health insurance, and the fact that health is influenced by various factors, empirical models cannot control for all influencing factors and may suffer from omitted variable bias. In this study, instrumental variable methods are used to correct for endogeneity. Following the approach used by relevant scholars, this study selects the “participation rate of urban-rural resident health insurance in other prefecture-level cities within the same province” as the instrumental variable (27–30). In terms of relevance, after the State Council released the “Opinions on Integrating the Basic health insurance Systems for Urban and Rural Residents” in 2016, various provinces successively issued their own policies for integrating the basic health insurance system for urban and rural residents, outlining integration schedules (31, 32). Provinces supervise and encourage cities at the prefecture-level to promote the integration of urban and rural resident health insurance. Under the governance logic of “competition among peers” and “elimination of the lowest performers,” the integration speed of other prefecture-level cities can naturally affect the integration progress in the prefecture-level city where rural older people individuals reside (33). ^.^In terms of exogeneity, the “participation rate of urban and rural resident health insurance in other prefecture-level cities in the same province as rural older people individuals” is unlikely to have a direct impact on the health of residents in the prefecture-level city where the older people individuals reside (34). It is not influenced in a reverse direction by the health of these residents and is also not correlated with individual or household characteristics. This satisfies the exogeneity condition for the instrumental variable (35).

Table 8 presents the estimation results for the two-stage 2SLS model. Since this study has only one endogenous explanatory variable and one instrument variable, there is no need for an over identification test. Therefore, this study first conducted the Durbin-Wu-Hausman test, and the results indicate that the estimated model in this study may suffer from endogeneity issues, with the explanatory variable “whether to participate in the integration of urban and rural resident health insurance” being an endogenous explanatory variable (36). Next, this study conducted a weak instrument test, and the results significantly rejected the null hypothesis of “weak instrument,” indicating no weak instrument problem. After adjusting for instrumental variables, urban-rural resident health insurance and integration still significantly reduced the frailty index, promoting the health of rural older people people (37). Compared to not controlling for endogeneity, controlling for endogeneity results in an increase in the absolute value of the regression coefficient for the variable “whether to participate in urban-rural resident health insurance.” If endogeneity is not addressed, the health effects of urban-rural resident health insurance integration will be underestimated (38).

**Table 8:** Instrumental variable analysis of the impact of urban and rural resident health insurance integration on health.

**2SLS**	
First-stage regression: IV	0.3095^***^
	(0.0384)
Second-stage regression: Urban and rural resident health insurance integration	−0.1302^***^
	(0.0309)
Control variables	Control
Weak instrument test	138.74
Durbin-Wu test	16.16^***^

Standard errors in parentheses, ^***^, ^**^, ^*^ indicate significance at the 1%, 5%, and 10% levels, respectively.

Furthermore, this study employs Instrumental Variable Quantile Regression (IVQR) to estimate different quantiles. As shown in Table 9, after adjusting for instrumental variables, the impact of healthcare integration on the frailty index remains consistent with the findings of the baseline regression, with differences observed across different quantiles of the frailty index (39). In quantile regression, at the 10, 25, 50, 75, and 90th quantiles, healthcare integration for urban and rural residents leads to decreases in the frailty index of 0.0835, 0.0956, 0.1150, 0.1434, and 0.1771, respectively. These effects are statistically significant at least at the 10% level. This also demonstrates that there is heterogeneity in the impact of healthcare integration for urban and rural residents on older people individuals in rural areas, with a greater health promotion effect observed among those with poorer health. Furthermore, after controlling for endogeneity, the absolute value of the coefficients increases (40). If endogeneity is not controlled for, it may result in an underestimation of the health impact of healthcare integration for urban and rural residents across different health quantiles (41–43).

**Table 9:** Instrumental variable quantile regression of the impact of urban and rural residents' healthcare integration on health.

	**Q10**	**Q25**	**Q50**	**Q75**	**Q90**
Whether experienced urban and rural residents' healthcare integration	−0.0835^*^	−0.0956^**^	−0.1150^***^	−0.1434^***^	−0.1771^***^
	(0.0466)	(0.0380)	(0.0283)	(0.0316)	(0.0547)
Control variables	Yes	Yes	Yes	Yes	Yes
Regional effects	Yes	Yes	Yes	Yes	Yes

Standard errors in parentheses, ^***^, ^**^, ^*^ indicate significance levels at 1%, 5%, and 10%, respectively.

#### 4.5.2 PSM-DID

**Figure 5:**
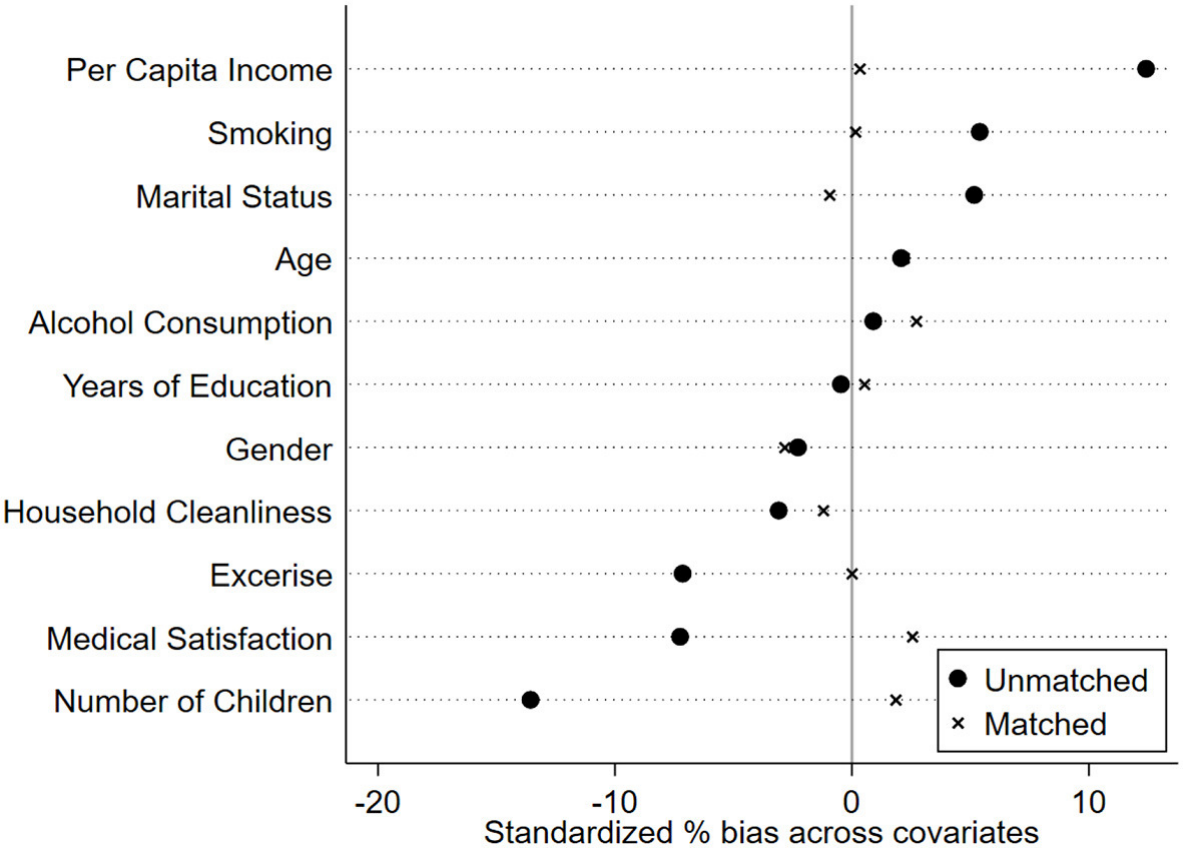
Analysis of PSM-DID matching results for the impact of urban-rural resident health insurance integration on health.

PSM-DID is an approach that integrates Propensity Score Matching (PSM) and Difference-in-Differences (DID) methods, which allows for a more comprehensive resolution of issues related to sample self-selection when dealing with the traditional DID framework. It effectively controls for sample bias under observable feature conditions. This study, as previously described, employed control variables and utilized the nearest neighbor matching method. After matching, there were no significant differences in the feature variables between the control group and the treatment group (as shown in Figure 5). This ensures that there were minimal significant differences between the control and treatment groups before the implementation of the urban-rural health insurance integration policy.

As shown in Table 10, after matching, whether it is the full sample or different quantiles, the coefficient of the impact of urban-rural resident health insurance integration on the frailty index remains negative, consistent with the baseline regression results, and still exhibits variations across different quantiles of the frailty index. In the full sample, urban-rural resident health insurance integration leads to a reduction of 0.0209 in the frailty index. In quantile regression, at the 10, 25, 50, 75, and 90th quantile points, urban-rural resident health insurance integration results in reductions of 0.0116, 0.0127, 0.0097, 0.0181, and 0.0280 in the frailty index, respectively, and is statistically significant at least at the 10% level.

**Table 10:** PSM-DID estimates of the impact of urban-rural resident health insurance integration on health.

	**Full sample**	**Q10**	**Q20**	**Q50**	**Q75**	**Q90**
DID	−0.0209^***^	−0.0116^***^	−0.0127^**^	−0.0097^*^	−0.0181^*^	−0.0280^*^
	(0.0067)	(0.0042)	(0.0052)	(0.0050)	(0.0095)	(0.0173)
Region effect	Yes	Yes	Yes	Yes	Yes	Yes
Control variables	Yes	Yes	Yes	Yes	Yes	Yes
Sample size	2,637					

Standard errors in parentheses, ^***^, ^**^, ^*^ indicate significance at the 1%, 5%, and 10% levels, respectively.

#### 4.5.3 Placebo test

To further control for the potential influence of unobservable characteristics on the impact of the urban-rural resident health insurance integration policy on rural older people individuals, this study conducted a placebo test. First, assuming that the large-scale rollout of the urban-rural resident health insurance integration in China did not occur in 2016 but in 2014. Second, selecting the data from two periods before the actual policy intervention in 2013 and 2015, using the same experimental and control groups, and reevaluating the impact of the urban-rural resident health insurance integration policy using the DID method. Table 11 results show that changing the policy timing did not yield significant results, indicating a low likelihood of time-varying heterogeneity effects. This supports the conclusion that the trend changes between the experimental and control groups are caused by the urban-rural resident health insurance integration.

**Table 11:** Placebo test of the impact of urban–rural resident health insurance integration on health.

	**Full sample**	**Q10**	**Q20**	**Q50**	**Q75**	**Q90**
DID	0.0069	0.00004	0.00066	−0.0068	−0.0089	0.0166
	(0.0092)	(0.00762)	(0.00536)	(0.0087)	(0.0096)	(0.0151)
Region effects	Yes	Yes	Yes	Yes	Yes	Yes
Control variables	Yes	Yes	Yes	Yes	Yes	Yes
Sample size	5,002					

Standard errors in parentheses, ^***^, ^**^, ^*^ denote significance levels at 1%, 5%, and 10%, respectively.

## 5 Discussion

The integration of urban and rural resident health insurance has been shown to improve residents' health outcomes, particularly for those with the lowest levels of health. After the integration of urban and rural resident health insurance in China, there exists a significant disparity in benefits between urban employee health insurance and urban and rural resident health insurance systems. This represents a critical focal point for future healthcare reform in China. Efforts should be made to gradually reduce the disparities in benefits between urban and rural resident health insurance and urban employee health insurance. The ultimate goal should be to achieve parity in benefits within the healthcare insurance system.

The reduction in healthcare burden promotes residents to seek medical care proactively when they fall ill, increase their investments in personal health, and enhance their nutritional intake. Therefore, in healthcare policy, it is advisable to reduce the barriers to seeking medical care, lower the deductible thresholds for medical visits, implement health poverty alleviation measures, establish a mechanism for the timely and accurate identification of assistance recipients, with a particular focus on identifying extremely impoverished individuals, older people citizens, people with disabilities, and low-income rural residents, among others. Gradually strengthening the construction of grassroots public health facilities, promoting the allocation of healthcare resources and financial funding to rural areas and the central and western regions, and addressing the development imbalance between urban and rural areas and among different regions.

The elevation of healthcare insurance coordination levels can encourage residents to seek medical treatment at higher-level hospitals, thereby obtaining better medical coverage, reducing the financial burden on patients, and alleviating their suffering. Gradually achieving provincial or even nationwide coordination of urban and rural residents' basic health insurance is an internal requirement for accelerating the integration and unification of healthcare insurance for residents. In September 2021, the State Council General Office issued the “Notice on Printing and Distributing the ‘14th Five-Year Plan' for National Medical Security,” further clarifying the goal of “raising the level of fund coordination” during the “14th Five-Year Plan” period. This goal includes “comprehensively implementing basic health insurance pooling at the city and prefecture levels based on the standards of unified institutional policies, pooled fund collection and expenditure, and integrated management services.” Additionally, it emphasizes the promotion of provincial-level pooling. These measures are expected to significantly improve the healthcare and medical security for a wide range of residents, including rural populations.

## 6 Conclusion

In terms of significance, this article further analyzes the health effects of medical insurance on people with different levels of health based on the Grossman model, enriching relevant academic research. From a practical perspective, Against the backdrop of an aging population and the imperative need to improve the health level of older adult people in rural areas, reducing the vulnerability of older adult people is not only an inherent requirement for the integration of urban and rural resident medical insurance systems, but also an inevitable choice to promote public medical and health services. This study used multiple rounds of data from the China Longitudinal Study on Health and Retirement (CHARLS) and employed the quantile double difference (DID) method to systematically examine the impact of integrated health insurance for urban and rural residents on the individual vulnerability of older adult people in rural areas.

The research results are summarized as follows. One is the integration of medical insurance for urban and rural residents, which alleviates the individual vulnerability of older adult people in rural areas and has a significant impact on older adult people with poor health conditions. Even after addressing endogeneity and conducting robustness testing, this conclusion still holds. Secondly, the health outcomes of the urban-rural medical insurance integration policy exhibit significant heterogeneity across different age groups and regions. Specifically, this policy has a more significant impact on the health outcomes of the older adult and residents in western regions. Thirdly, the integration of medical insurance for urban and rural residents plays a role by reducing the medical burden mechanism, thereby improving health and alleviating vulnerability. In addition, reducing the medical burden has a more significant impact on individuals with lower health conditions. In summary, developing countries like China should gradually narrow the welfare gap between different medical insurance systems, implement medical poverty alleviation measures, reduce the medical burden on impoverished populations, and continue to strengthen the coordination of medical insurance programs for urban and rural residents.

However, due to data limitations, this article also has some limitations. Firstly, we only analyzed the impact of medical insurance on rural middle-aged and older adult people, while medical insurance also plays an extremely important role in other vulnerable groups such as children and women. In the future, research is needed on the impact of different populations. Secondly, this article mainly analyzes how medical insurance affects the proportion of medical reimbursements, resulting in different health effects for different healthy populations. In the future, other data should be used to analyze different impact pathways.

## Data availability statement

The original contributions presented in the study are included in the article/supplementary material, further inquiries can be directed to the corresponding author.

## Ethics statement

The studies involving humans were approved by China Health and Retirement Longitudinal Study. The studies were conducted in accordance with the local legislation and institutional requirements. The participants provided their written informed consent to participate in this study. Written informed consent was obtained from the individual(s) for the publication of any potentially identifiable images or data included in this article.

## Author contributions

CQ: Writing – original draft. XW: Writing – review & editing.

## Footnotes

^1^Depression level is assessed based on whether you experienced the following symptoms over the past week: “difficulty sleeping, feeling easily annoyed or irritated, trouble concentrating on things, feeling everything is an effort, feeling downhearted, frightened, or lonely,” as well as “feeling happy and hopeful about the future.” Each symptom is scored on a scale of 0–3, with a frequency of < 1 day scoring 0, 1–2 days scoring 1, 3–4 days scoring 2, and 5–7 days scoring 3. However, for the items “feeling happy” and “feeling hopeful about the future,” the scoring is reversed. The scores are then standardized to a range of 0–1.
